# Retention Mechanisms of Citric Acid in Ternary Kaolinite-Fe(III)-Citrate Acid Systems Using Fe K-edge EXAFS and L_3,2_-edge XANES Spectroscopy

**DOI:** 10.1038/srep26127

**Published:** 2016-05-23

**Authors:** Jianjun Yang, Jian Wang, Weinan Pan, Tom Regier, Yongfeng Hu, Cornelia Rumpel, Nanthi Bolan, Donald Sparks

**Affiliations:** 1 Department of Plant and Soil Sciences, Delaware Environmental Institute, University of Delaware, Newark, USA, 19716; 2 Canadian Light Source Inc., University of Saskatchewan, Saskatoon, Canada, S7N 2V3; 3 CNRS, Institute of Ecology and Environment Paris, IEES, UMR (CNRS-INRA-UPMC-UPEC-IRD), Thiverval-Grignon, France, 78850; 4 Global Centre for Environmental Remediation (GCER), University of Newcastle, NSW, 2308, Australia; 5 Centre for Environmental Risk Assessment and Remediation (CERAR),University of South Australia, Mawson Lakes, Australia, SA 5095

## Abstract

Organic carbon (OC) stability in tropical soils is strongly interlinked with multivalent cation interaction and mineral association. Low molecular weight organic acids (LMWOAs) represent the readily biodegradable OC. Therefore, investigating retention mechanisms of LMWOAs in mineral-cation-LMWOAs systems is critical to understanding soil C cycling. Given the general acidic conditions and dominance of kaolinite in tropical soils, we investigated the retention mechanisms of citric acid (CA) in kaolinite-Fe(III)-CA systems with various Fe/CA molar ratios at pH ~3.5 using Fe K-edge EXAFS and L_3,2_-edge XANES techniques. With Fe/CA molar ratios >2, the formed ferrihydrite mainly contributed to CA retention through adsorption and/or coprecipitation. With Fe/CA molar ratios from 2 to 0.5, ternary complexation of CA to kaolinite via a five-coordinated Fe(III) bridge retained higher CA than ferrihydrite-induced adsorption and/or coprecipitation. With Fe/CA molar ratios ≤0.5, kaolinite-Fe(III)-citrate complexation preferentially occurred, but less CA was retained than via outer-sphere kaolinite-CA complexation. This study highlighted the significant impact of varied Fe/CA molar ratios on CA retention mechanisms in kaolinite-Fe(III)-CA systems under acidic conditions, and clearly showed the important contribution of Fe-bridged ternary complexation on CA retention. These findings will enhance our understanding of the dynamics of CA and other LMWOAs in tropical soils.

Low molecular weight organic acids (LMWOAs) are prevalent in soil systems and form an important labile C source for microorganisms^1^. Although LMWOAs are typically found in relatively low concentrations (<50 μM) in the soil solution, the extremely rapid mineralization rate of LMWOAs (mean residence time 1–10 h) could contribute to as high as 30% of the total soil CO_2_ flux^2^. The association of LMWOAs with minerals has been recognized as one of the major mechanisms for increasing LMWOAs stability against microbial degradation^1^,^3^,^4^,^5^. Therefore, alterations in the retained amount and binding structure of LMWOAs are of significant importance in soil C stability, dynamics and cycling.

Tropical soils generally support forest and woodland systems which preserve a large C pool^6^, thus playing a vital role in global C cycling. Many tropical soils are highly weathered and acidic (3.0~5.0)^7^,^8^, and their clay mineralogy is generally dominated by kaolinite^9^,^10^,^11^,^12^. The low pH of tropical soils helps maintain a relatively high concentration of multivalent cations in the soil solution, with Fe^3+^ and Al^3+^ typically ranging from several to hundreds of μM, depending on specific soil properties^13^,^14^,^15^,^16^. Consequently, ternary systems that include kaolinite, metal cations and LMWOAs are prevalent in tropical soils. Given the possible co-existence of LMWOAs adsorption and ternary complexation via cation or ligand bridging on the mineral surfaces^17^,^18^, the retention and binding structure of LMWOAs in ternary kaolinite-cation-LMWOAs systems could be different compared to binary kaolinite- LMWOAs systems, thus deserving close attention.

Adsorption of LMWOAs on kaolinite has been widely studied^3^,^17^,^19^,^20^. For example, kaolinite exhibited a higher adsorption capacity for citric acid (CA) than 2:1 phyllosilicates (e.g illite)^3^,^17^,^19^ and outer-sphere complexation was proposed for the adsorption of CA on kaolinite within the pH range of 3.0 to 6.0 17,20; Yeasmin *et al.*^19^ also reported the co-existence of inner-sphere and outer-sphere complexation of CA on kaolinite above pH 6.0 using ATR-FTIR spectroscopy. However, the impact of interactions among LMWOAs, metal cations and minerals on the retention and binding mechanisms of LMWOAs on mineral surfaces is poorly understood. Varadachari *et al.*^21^ found significant increases in retention of non-water extractable humic acid (HA) on mineral surfaces in HA-montmorillonite/kaolinite/illite systems via cation bridging that included Al^3+^ and Ca^2+^. Ahmed *et al.*^22^ also indicated the significance of cation bridging in humus retention on clay minerals using citrate, EDTA, and oxalate extractions. It was observed that Al^3+^ and Fe^3+/2+^ rather than Ca^2+^ mainly contributed to the binding of clay and humus via cation bridging in an Alfisol soil (pH 5.40). These results agreed with the less effectiveness of bivalent compared to trivalent Al^3+^ and Fe^3+^ in cation bridging to clay minerals under acidic conditions^16^. Compared to humic acid or humus with relatively high aromatic-C^23^,^24^, LMWOAs were enriched with aliphatic- and carboxylic-C, which probably resulted in the different retention capacity and binding structure of LMWOAs on minerals in ternary mineral-cation-LMWOAs systems.

The phenomenon that multivalent cations enhance the sorption of OC to mineral surfaces is well-known^16^,^25^, but only a few studies address the sorption mechanisms at the molecular scale^18^. Of all multivalent cations in acidic soil solutions, Fe^3+^ and Al^3+^ are regarded as the most important candidates to form cation bridges between OC and minerals^13^,^16^,^22^. Synchrotron-based X-ray absorption spectroscopy (XAS), including X-ray absorption near-edge structure (XANES) and extended X-ray absorption fine structure (EXAFS) spectroscopy, has been recognized as the most promising technique to characterize the molecular speciation of elements of interest^26^. For example, Fe K-edge EXAFS spectroscopy has been widely used to characterize Fe speciation in soils, surface water and mineral-organic acid systems^27^,^28^,^29^,^30^,^31^. Recently, Fe L_3,2_-edge XANES spectroscopy, arising from dipole-allowed Fe 2p→3d electron transitions, demonstrates great advantage in probing Fe-ligand interactions, because this technique not only has high-intensity and fine spectral features but also is sensitive to ligand fields surrounding the Fe 3d orbital compared to K-edge XANES^32^,^33^. However, it is difficult to use XAS to speciate structural Al in sorption samples from kaolinite-Al-LMWOAs systems since the Al in kaolinite displays the predominant XAS signal. Consequently, it is hard to spectrally deconvolute ternary complexed Al that is of relatively low concentration on the kaolinite surface. Furthermore, the EXAFS spectra of Al, as a light element, are difficult to measure using soft X-rays for sorption samples, primarily due to the relative low Al content. This could account for Al EXAFS data existing only for pure compounds like Al oxides^34^. Based on these reasons, Fe(III) was chosen to investigate the retention and binding mechanisms of LMWOAs in ternary kaolinite-cation-LMWOAs systems.

In this study, CA was selected to represent LMWOAs because it is one of the major constituents in root exudates^35^ and microbial metabolites^36^ and is widely distributed in the soil solution^1^,^37^. Batch experiments were conducted using model systems of kaolinite and CA with and without Fe(III) under acidic conditions, and the sorption products were characterized by Fe K-edge EXAFS and L_3,2_-edge XANES spectroscopy. Accordingly, the objectives of this study are: 1) to compare the retention of CA between binary kaolinite-CA and ternary kaolinite-Fe(III)-CA systems; and 2) to reveal the retention mechanisms of CA and their relative contribution in ternary systems with various Fe/CA molar ratios.

## Results

### Enhanced CA retention in kaolinite-Fe(III)-CA system

CA retention in the ternary kaolinite-Fe(III)-CA systems was generally higher than that in the binary kaolinite-CA systems under acidic conditions (Table 1). Depending on the CA concentration added, sorbed CA ranged from 2.470 to 195.2 mg g^−1^ kaolinite, accounting for 15.84 to 64.29% of the total added CA, in kaolinite-CA systems. This increased from 3.842 to 212.0 mg g^−1^ kaolinite, with percentages of 25.8 to 100% total added CA, in the kaolinite-Fe(III)-CA systems. Obviously, the addition of Fe^3+^ significantly enhanced CA retention in the kaolinite-CA systems. Furthermore, maximum CA retention in the ternary systems occurred at an Fe/CA molar ratio of 10 (Table 1). The corresponding sorption sample S1 contained the maximum retained Fe (Table 1 and S1). Although the kaolinite-Fe(III)-CA systems had maximum CA retention at an Fe/CA molar ratio of 10, the highest percentage (195.8%) of increase in CA in the kaolinite-Fe(III)-CA systems relative to the kaolinite-CA systems occurred at an Fe/CA molar ratio of 2 (Table 1). Within the Fe/CA molar ratios ranging from 0.25 to 0.05, the enhanced amounts of CA retained in the kaolinite-Fe(III)-CA systems relative to the kaolinite-CA systems reached a maximum and displayed insignificant variation around 15.21~16.82 mg g^−1^ kaolinite. This indicated that Fe(III)-induced CA retention remained unchanged in the ternary systems with Fe/CA molar ratios from 0.25 to 0.05. Additionally, solution pH remained almost constant in the kaolinite-CA systems but significantly decreased in the kaolinite-Fe(III)-CA systems with an initial CA concentration greater than 0.25 mM as compared to the kaolinite-CA systems (Table S1). The unchanged solution pH of the kaolinite-CA systems could suggest outer-sphere binding of CA on the kaolinite surface under acidic conditions^17^,^20^. However, the reduced solution pH of the kaolinite-Fe(III)-CA systems probably resulted from the consumption of hydroxyl ions when Fe hydroxides formed and/or the release of protons due to complexation of CA to Fe^3+^ and inner-sphere complexation of CA or a soluble Fe(III) citrate complex on the mineral surface. According to Lackovic and his co-authors’ study^17^, such a minor pH decrease (<0.2) around pH 3.5 had little impact on the adsorption of CA on kaolinite. Therefore, the addition of Fe(III) mainly resulted in the increased CA retention in the kaolinite-Fe(III)-CA systems relative to the kaolinite-CA systems.

### Fe EXAFS spectroscopic analysis

**Figure 1:**
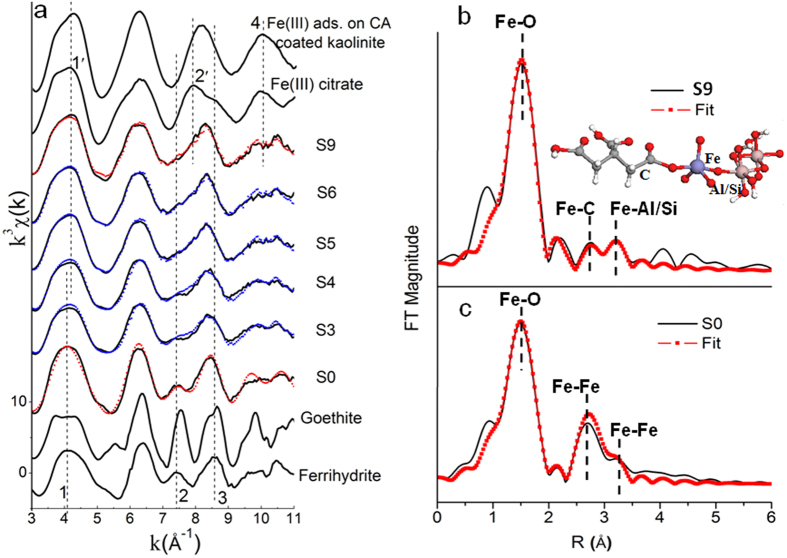
EXAFS spectroscopic analysis of Fe speciation in selected sorption samples from the kaolinite-Fe(III)-citrate system: k^3^-weighted Fe K-edge EXAFS spectra. (**a**) The Fourier transform magnitude of S9 (**b**) and S0 (**c**). S0, S3 to S6 and S9 represent sorption samples without CA addition and with the ratio of Fe/citrate acid as 2, 1, 0.5, 0.25 and 0.05, respectively. Red and blue dotted lines represent the results obtained from shell-fitting and linear combination fitting, respectively. Peak features of interest are labeled by numbers 1 to 4.

Multiple Fe forms were expected to exist in the ternary systems and responsible for the enhanced CA retention under varied Fe/CA molar ratios. The possible Fe species included ternary kaolinite-Fe(III)-citrate and kaolinite-citrate-Fe(III) complexes from ternary complexation via Fe or ligand bridges^18^,^38^, Fe hydroxides (e.g. ferrihydrite) or Fe hydroxide-CA coprecipitates due to Fe hydrolysis^39^,^40^ as well as an Fe citrate precipitate^4^. Without CA addition, the Fe K-edge EXAFS spectra of sorption sample S0 from the kaolinite-Fe(III) system showed similar peak features at ~4.0 Å^−1^ (peak 1), ~7.5 Å^−1^ (peak 2), ~8.5 Å^−1^ (peak 3) and 10 Å^−1^ (peak 4) to ferrihydrite rather than goethite (Fig. 1a), indicating the formation of less crystalline ferrihydrite in sample S0. Shell-fitting analysis of the S0 EXAFS spectra indicated Fe had 5.3 Fe-O bonds with an average distance of 1.98 Å in the first shell, and coordinated with 1.8 and 2.5 Fe atoms at average distances of 3.13 Å and 3.38 Å in the second and third shell, respectively (Table 2). The first-shell coordination number of Fe in the S0 is reasonable given that ferrihydrite has both tetrahedral (four-coordinated) and octahedral (six-coordinated) Fe(III)^31^,^41^. Other structural parameters of Fe in the S0 sample were consistent with those of the reported ferrihydrite^28^,^31^,^42^, thus confirming the formation of Fe precipitates as ferrihydrite in sample S0.

At the high Fe/CA molar ratio, ferrihydrite, ferrihydrite-CA coprecipitates and Fe citrate precipitates probably dominated in kaolinite-Fe(III)-citrate systems under acidic conditions. For the sample S3 at a Fe/CA molar ratio of 2, the peak 2 at ~7.5 Å^−1^, is a characteristic peak of ferrihydrite^31^, and became less evident in the Fe EXAFS spectra. Peak 3 at ~8.5 Å^−1^ shifted to the low k side, though the peak 1 position at ~4.0 Å^−1^ remained unchanged, as compared to the S0 and ferrihydrite reference (Fig. 1a). When further decreasing the Fe/CA molar ratio from 1 to 0.05, peak 2 disappeared in the spectra of samples S5, S6 and S9, while peak 1 at ~4.0 Å^−1^ and peak 3 at ~8.5 Å^−1^ of these samples shifted to high and low k sides, respectively. These results strongly indicated the dissolution of ferrihydrite with the increased CA addition, and the transformation of aqueous Fe^3+^ to ferrihydrite was mainly retarded with Fe/CA molar ratios less than 0.5. Consistently, gradual decreases in the magnitude of the second shell (Fe-Fe pair) were observed in the Fe K-edge Fourier transformed (FT) EXAFS spectra of sorption samples when the Fe/CA molar ratio decreased from 2 to 0.05 (Figure S1). This suggested the loss of a second-shell coordinated Fe structure. Mikutta *et al.* also reported reduced intensity of the peak at ~7.5 Å^−1^ in the Fe K-edge EXAFS spectra as a result of the dissolution of ferrihydrite induced by hydroxybenzoic acids^31^. The lack of predominate peak 2´ at ~8.0 Å in the spectra of S3, S4, S5, S6 and S9 indicated the absence of solid Fe(III) citrate in these samples (Fig. 1a), thus qualitatively excluding the formation of an Fe(III) citrate precipitate in kaolinite-Fe(III)-CA systems.

At the low Fe/CA molar ratio, the retained Fe on kaolinite was expected to dominate as ternary kaolinite-Fe(III)-citrate and/or kaolinite-citrate-Fe(III) complexes. For the selected sample S9 obtained at an Fe/CA molar ratio of 0.05, a ternary kaolinite-Fe(III)-citrate complex is the preferentially targeted Fe species retained on kaolinite according to the EXAFS analysis (Fig. 1a,b, Table 2). In the EXAFS spectra of ferrihydrite, the beat pattern in the Fe EXAFS spectra at 10 Å^−1^ were attributed to third-shell Fe scattering^31^,^39^. Compared to the ferrihydrite reference, the absence of beat patterns in the EXAFS spectra at 10 Å^−1^ for Fe(III) citrate and Fe(III) adsorbed on CA coated kaolinite suggested the lack of significant third-shell scattering. This agreed with the complexation of Fe by carboxylic groups of citrate in these two references which only had C/O atoms at the third shell of Fe. Consistently, the observed multiple scatter peaks due to a Fe–C–O structure^43^,^44^ (Figure S1) also indicated the association of Fe with carboxylic groups of citrate in Fe(III) citrate and Fe(III) adsorbed on CA coated kaolinite reference samples. However, the beat pattern at 10 Å^−1^ was observed in the Fe EXAFS spectra of S9, which excluded the dominant presence of kaolinite-CA-Fe(III) complexes in S9. As there was little ferrihydrite in S9 due to the high CA loading (Table 1 and Fig. 1a), Al/Si atoms in kaolinite were the only potential candidates as the third-shell backscattering atoms of Fe in S9, and thus ternary complexation of kaolinite and CA via an Fe(III) bridge was probably the primary mechanism for CA retention in S9 (Fig. 1b).

Shell-fitting analysis further confirmed the formation of a ternary kaolinite-Fe(III)-citrate complex in S9. The best fitting results indicated that the first shell of Fe(III) was coordinated with 5 O atoms with an average Fe–O distance of 2.00 Å, which is in fair agreement with other reported Fe-O bond lengths of organic Fe(III) complexes^29^. However, the 5 coordination number of Fe in the S9 sample was lower than the octahedral coordination previously reported for Fe(III) in organic Fe(III) complexes^29^,^43^. Beyond the first Fe–O shell, the second shell could be modeled with 1.0 C atom at 3.16 Å and a third shell with 1.0 Al/Si atoms at 3.40 Å. In the second shell, the formation of a monodentate-mononuclear structure between Fe(III) ions and citrate groups probably resulted from the low pH conditions (~pH 3.5) under which only one carboxylic group of CA (pKa1 = 3.14, pKa2 = 4.75) was primarily deprotonated. Thus, a little longer Fe-C distance (~3.16 Å) was expected compared to the reported 2.72~3.0 Å in soil organic Fe complexes where Fe was more firmly chelated and formed multiple bonds with organic functional groups^26^,^28^. For the third Fe-Al/Si shell, few studies have been reported on the local structure of Fe(III) adsorbed on kaolinite. However, our fitting results for the third Fe-Al/Si shell agreed well with the reported structures of cobalt (Co) adsorbed on kaolinite (Co-Al/Si distance ranging from 3.38~3.43 Å)^45^, since there are similar atomic radii and chemical properties for Fe(III) and Co. Similar distances of the Fe-Al/Si pair (2.8~3.4 Å) were also reported for zeolite containing Fe^46^. Therefore, a molecular structure of a five-coordinated Fe(III) bridge between kaolinite and CA was proposed (Fig. 1b), which could well account for the increased CA retention in the kaolinite-Fe(III)-CA systems compared to the kaolinite-CA systems. According to our proposed Fe(III) model for sample S9, the total adsorbed Fe on kaolinite (0.079 mM g^−1^ kaolinite) should contribute to the same quantity of retained CA, 0.079 mM g^−1 ^kaolinite (Table S2). This accounted for 89.8% of the total enhanced CA (0.088 mM g^−1^ kaolinite) derived from batch experiments on the kaolinite-Fe(III)-CA system relative to the kaolinite-CA system (Table S2). These results are reasonable because the 10.2% discrepancy in the retained CA probably resulted from the presence of minor soluble Al^3+^ which could also form a ternary kaolinite-Al(III)-citrate complex^18^,^21^. Therefore, our proposed ternary complexation model of Fe(III) bridged between kaolinite and CA based on molecular-level XAS analysis (Fig. 1b) was further validated. A similar ternary complexation model via a six-coordinated Fe(III) in goethite-oxalate systems was also proposed under acidic conditions by Simanova *et al.*^18^ using FTIR and Ga(III) K-edge EXAFS spectroscopy. However, due to the lack of direct Fe K-edge EXAFS and L_3,2_-edge XANES analysis, it is hard to exclude the five-coordinated Fe(III) in their systems considering the similar surface reaction characteristics of kaolinite to goethite^45^.

Within the medium Fe/CA molar ratio, the incomplete dissolution of ferrihydrite and the presence of certain soluble Fe citrate complexes probably resulted in the co-existence of ferrihydrite and a ternary kaolinite-Fe(III)-citrate complex in sorption samples. Principle component analysis revealed two significant Fe components in the selected sorption samples S3 to S6, with Fe/CA molar ratios ranging from 2 to 0.25 (Table S3). Target transformation analysis indicated ferrihydrite and a ternary kaolinite-Fe(III)-citrate complex were the most possible end-members for these samples (Table S4). In agreement, both ferrihydrite and a ternary kaolinite-Fe(III)-citrate complex were also targeted as significant Fe components by LCF analysis (Table 2). LCF results indicated the proportion of ternary complexed Fe species increased from 17% to 85% but that of ferrihydrite decreased from 83% to 15% when decreasing the Fe/CA molar ratio from 2 to 0.25 (Table 2). According to the molecular structure of the kaolinite-Fe(III)-citrate complex (Fig. 1b), each Fe atom could bind one CA molecule to form this ternary complex. As the retained ternary complexed Fe in the kaolinite-Fe(III)-CA systems ranged from 4.555 to 18.83 μmol, the bound CA in the kaolinite-Fe(III)-citrate complex should have the same molar amount and thus could account for 46.44% to 82.75% of the total retained CA (Table S5). Therefore, ternary complexation via an Fe bridge retained 56.89% to 82.75% of the total initial CA on the kaolinite surface with Fe/CA molar ratios from 2 to 0.5. This strongly indicated a higher contribution due to ternary complexation than ferrihydrite-induced adsorption and/or coprecipitation on CA retention in the kaolinite-Fe(III)-CA systems. The predominate contribution of ternary complexation via an Fe bridge rather than adsorption or coprecipitation for CA retention has been little reported previously, although such medium Fe/CA molar ratios (2 to 0.5) widely occur in the acidic soil solutions of tropical soils^16^,^37^. At an Fe/CA molar ratio of 0.5, the reduced CA retention (46.44%) by Fe-bridged ternary complexation probably resulted from the following two reasons: 1) the ternary complexed CA reached a maximum (16.55 mg g^−1^ kaolinite) as a result of limited initial Fe addition; and 2) the total retained CA on kaolinite significantly increased (51.92 g^−1^ kaolinite) in the binary kaolinite-CA system with a high initial CA concentration of 4 mM.

### Fe L_3,2_-edge XANES analysis

**Figure 2:**
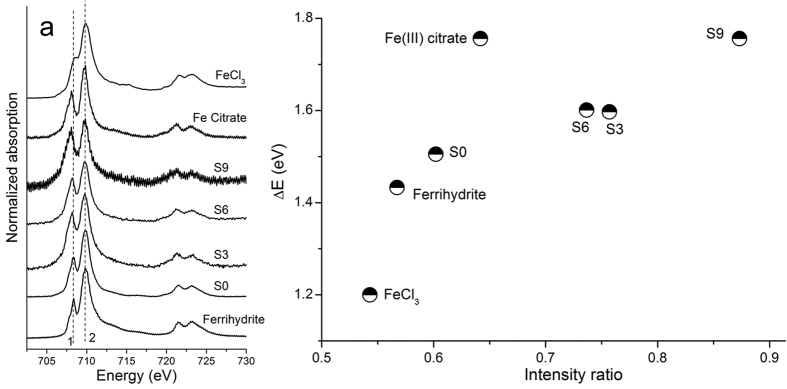
Fe L_3,2_-edge XANES spectra (**a**) and the corresponding Fe L_3_-edge peak intensity ratio vs peak energy difference (ΔE) characterization plot (**b**) of the selected Fe references and sorption samples from the kaolinite-Fe(III)-citric acid (CA) system. S0, S3, S6 and S9 represent sorption samples without CA and with Fe/CA molar ratios of 2, 0.5 and 0.05. Peak features of interest are labeled by numbers 1 to 2.

Fe L_3,2_-edge XANES probes the dipole allowed transition of a Fe 2p electron to the unoccupied 3d orbital which partially contributed to the valence molecular orbital in the organic Fe complex, thus being sensitive to the covalency of Fe-ligand bonds^47^. Due to spin-orbital coupling, two main spectral features were resolved at the 706–713 eV (L_3_-edge) and 720–725 eV (L_2_-edge) regions for all the selected sorption samples (Fig. 2a). Furthermore, an additional splitting of these two main peaks, arising from metal-ligand electronic interactions, was also observed (Fig. 2a).

Compared to ferrihydrite, the energy position and intensity of Fe L_3_-edge peaks 1 and 2 of sorption sample S0 exhibited invisible changes (Fig. 2a,b), which further validated the EXAFS results that the dominance of Fe existed as ferrihydrite in sample S0. However, the energy position of peak 1 in sample S9 shifted to the low-energy side relative to ferrihydrite and other samples including S0, S3 and S6, and was finally aligned to the Fe citrate reference (Fig. 2a,b). Simultaneously, there was a visible increase in the intensity of the Fe L_3_-edge peak 1 of sample S9 compared to ferrihydrite and sample S0. Hocking *et al.* attributed the low-energy-side shift of the Fe L_3_-edge peak 1 and its increased peak intensity to the enhanced covalency of the Fe-ligand bond in organic Fe complexes according to theoretical calculations^48^. However, in our study, the kaolinite-Fe(III)-citrate complex in sample S9 had the same ligand element (oxygen) as ferrihydrite, and only one carboxylic functional group was bound with Fe as monodentate-mononuclear structure under the investigated acidic conditions. Therefore, the Fe-ligand covalency seems to be not strong enough to induce the significantly changed energy position and intensity of peak 1 in the Fe L_3,2_-edge XANES spectra of sample S9 compared to ferrihydrite. von der Heyden *et al.*^49^ reported a summarized L_3_-edge peak intensity ratio for a variety of pure Fe (III) compounds with different ligands, and indicated that the peak intensity ratio of both inorganic and organic Fe (III) compounds with six Fe-O bonds (octahedral structure) in the first shell ranged from 0.376 (Fe_2_(MO_4_)_3_) to 0.766 (goethite), which was much less than the peak intensity ratio of 0.873 for sample S9. These results further implied the limited impact of Fe-O covalency on the changes of the L_3_-edge peak intensity ratio in the Fe L_3,2_-edge XANES spectra of sample S9.

Alternatively, the altered Fe first-shell coordination structure probably mainly accounted for the aforementioned peak intensity changes for sample S9 (Table 2), because the core excitations at the Fe L-edge are highly localized to the first coordination sphere of Fe^50^, and thus the intensities of the Fe L_3_-edge peaks are very sensitive to the variation in Fe coordination^49^, particularly in the first shell. Consequently, the altered intensity of peak 1 in the S9 spectra probably reflected the five-coordinated structure in the first shell of Fe in the kaolinite-Fe(III)-citrate complex. This was because the five-coordinated Fe had a trigonal bipyramidal structure (D3h symmetry) with two classes of an unoccupied d-orbital (e′ and a_1_′, Figure S2)^51^, as indicated by the splitting of two L_3_-edge peaks in the Fe XANES spectra of sample S9 (Fig. 2a). The degree of the un-occupancy is larger for the low-energy state (e´) but smaller for the high-energy states (a_1_´) in the trigonal bipyramidal Fe(III) compared to those (low-energy state, t_2g_; high-energy state, e_g_) in octahedral Fe(III) (Figure S2). Therefore, we observed a relatively higher intensity of peak 1 but a lower intensity of peak 2 for sample S9 relative to ferrihydrite (Fig. 2a,b), dominant as an octahedral Fe(III) structure (O_h_ symmetry, Figure S2)^33^,^41^. Consequently, the Fe L_3,2_-edge XANES and K-edge EXAFS results supported the presence of a trigonal bipyramidal Fe(III) bridged between kaolinite and CA in sample S9.

## Discussion

With the Fe/CA molar ratios from 10 to 0.25, the added Fe partially transformed into ferrihydrite in kaolinite-Fe(III)-CA systems according to Fe K-edge EXAFS and L_3,2_-edge XANES analysis (Figs 1 and 2). The lack of measurable Fe citrate in sorption samples with Fe/CA molar ratios from 2 to 0.25 (S3 to S6), using EXAFS spectroscopy (Table 2), excluded precipitation of insoluble Fe citrate as one significant mechanism for CA retention in the investigated ternary systems. This is different from the study conducted by Chen *et al.* (2014)^52^, which showed the significant contribution of Fe-DOM (dissolved organic matter) precipitation on DOM retention with a C/Fe molar ratio more than 17.5 52. This probably resulted from the relatively high molecular weight of DOM and the lower solubility of Fe-DOM than Fe citrate, and thus it could more easily precipitate form the solution phase. In this study, the resulting ferrihydirte may serve as a new sorbent for CA retention and/or form kaolinite-ferrihydrite-CA coprecipitates. However, ferrihydrite-induced adsorption and coprecipitation mechanisms for CA retention were hard to differentiate in a single ternary system of this study, thus deserving further investigation.

The relative contribution of ferrihydrite-induced adsorption/coprecipitation and ternary complexation via Fe bridging on CA retention in ternary kaolinite-Fe(III)-CA systems was highly dependent on the Fe/CA molar ratios under acidic conditions. With a Fe/CA molar ratio >2, the majority of Fe transformed to ferrihydrite which acted as the major driving force for increased CA retention in the ternary kaolinite-Fe(III)-CA systems, either through adsorption by ferrihydrite or coprecipitation as a kaolinite-ferrihydrite-CA complex. The higher retention of CA on ferrihydrite than on kaolinite has been frequently reported^3^,^19^, but the impact of ferrihydrite-CA coprecipitation on CA retention has not been well documented, although previous studies indicated CA coprecipitation could influence the crystal structure and surface properties of the resulting Fe oxides^40^. Because ferrihydrite-DOM coprecipitation could retain at least equal OC compared to DOM adsorption on ferrihydrite^52^,^53^, it is reasonable to infer that ferrihydrite-induced adsorption and/or coprecipitation of CA mainly contributed to the increased retention of CA in the kaolinite-Fe(III)-CA systems relative to the kaolinite-CA systems with Fe/CA molar ratios >2. Therefore, ferrihydrite-induced adsorption and/or coprecipitation probably accounted for the observed highest retention of CA in the ternary systems with low CA loading (Table 1) due to limited complexation of Fe by CA, which prevented Fe hydrolysis. This was also supported by the similar adsorption isotherm pattern of CA in kaolinite-Fe(III)-CA systems with a Fe/CA molar ratio >2 (Figure S3) compared to those of DOM adsorbed on ferrihydrite and coprecipitated with ferrihydrite^52^.

When the Fe/CA molar ratio varied between 2 and 0.5, a ternary complex between CA and kaolinite via a Fe bridge accounted for 56.89~82.75% of the total retained CA in kaolinite-Fe(III)-CA systems (Table S5). This showed the higher contribution of Fe-bridged ternary complexation to the increased CA retention than ferrihydrite-induced adsorption and/or coprecipitation. Although the retention of OC by ferrihydrite through adsorption/coprecipitation and ternary complexation of OC on minerals via an Fe bridge were all previously shown^18^,^21^,^52^,^53^, few studies have been conducted to investigate the relative contribution of ferrihydrite-induced adsorption/coprecipitation and ternary complexation on OC retention in ternary mineral-OC systems. This study is the first to provide direct information to quantify the significant contribution of ternary complexation via Fe bridging on CA retention in mineral-OC systems under acidic conditions. As these Fe/CA molar ratios (2 to 0.5) are common in soil solutions of acidic tropical soils^16^, ternary complexation of CA or other LMWOAs via Fe bridging probably plays a significant role in C stability, dynamics and soil respiration. Hobbie *et al.*^16^ reported that the percentage of exchangeable Fe and Al was negatively correlated with soil respiration in acidic tropical soils and interpreted ternary complexation via cation bridging as one of the major contributing factors. As LMWOAs are readily biodegradable, Fe-bridge ternary complexation of LMWOAs on mineral surfaces probably increases the stability of LMWOAs against microbial degradation, thus partially accounting for the observed negative correlation between exchangeable Fe/Al and soil respiration. Furthermore, the highest degree of the increased CA retention in the kaolinite-Fe(III)-CA system relative to the kaolinite-CA system occurred at an Fe/CA molar ratio of 2 (Table 1), which indicated that compositions of ferrihydrite and ternary complexed Fe(III) at percentages of 83% and 17%, respectively, could most efficiently enhance CA retention in the ternary system. This provided insights on management regimes to effectively enhance CA or other LMWOAs retention in tropical soils.

Once the Fe/CA molar ratio was lower than 0.5, ferrihydrite formation was completely retarded and thus CA adsorption on kaolinite played a major role in CA retention in the kaolinite-Fe(III)-CA systems (Table 1). However, almost all the retained Fe on kaolinite existed as a ternary kaolinite-Fe(III)-citrate complex (Table 2). Under such a scenario, a soluble Fe-citrate complex and citrate acid competed for the binding sites on kaolinite. As inner-sphere complexation between kaolinite and the Fe-citrate complex via Fe bridging formed stronger bonds than outer-sphere complexation between kaolinite and CA^17^,^20^, the Fe-citrate complex should be preferentially associated with kaolinite rather than CA. Thus, the subsequent kaolinite-Fe-citrate complex would be more stable than the outer-sphere kaolinite-citrate complex against desorption and biodegradation. Therefore, whether adsorption or ternary complexation represented the major contributor for CA retention at Fe/CA molar ratios ≤0.5 depended greatly on the initial Fe concentration. With a higher initial Fe concentration, the higher proportion of an inner-sphere complexed Fe citrate complex would preferentially occupy the binding sites of kaolinite. This probably decreased the surface charge of kaolinite and thus less CA would be retained on the kaolinite surface via outer-sphere complexation in kaolinite-Fe(III)-CA systems with a Fe/CA molar ratio less than 0.5.

Although the retention mechanisms of CA and other LMWOAs in binary mineral-LMWOAs systems were intensively investigated both at the macro and molecular levels^3^,^17^,^19^,^20^, few studies have focused on the retention mechanisms of LMWOAs in ternary mineral-cation-LMWOAs systems which are widely distributed in acidic soils^16^. In this study, we demonstrated the significant impact of varying Fe/CA molar ratios on the retention and binding mechanisms of CA in a ternary kaolinite-Fe(III)-CA system under acidic conditions using multiple molecular-level techniques including Fe K-edge EXAFS and L_3,2_-edge XANES spectroscopy. Given the wide range in the Fe/CA molar ratio^16^,^54^ and the fast degradation rate of CA in various tropical soils^16^, all the aforementioned mechanisms for CA retention might occur under natural conditions. As the temperature of natural soils is generally higher than 4 °C used in this study, the corresponding sorption affinity of CA on kaolinite and/or ferrihydrite in the studied ternary systems may slightly decrease under natural conditions considering the limited negative impact of temperature increase on DOM sorption affinity on mineral surface^55^. The relative contribution of each aforementioned mechanism for CA retention depended greatly on the specific Fe/CA molar ratio as well as the Fe/kaolinite weight ratio. Because LMWOAs are generally enriched with carboxylic functional groups like CA, the binding mechanisms at high, medium and low Fe/CA ratios may also contribute to the retention of other LMWOAs in ternary kaolinite-Fe(III)- LMWOAs systems under acidic conditions. These findings are critical to understanding C stability, dynamics and cycling in acidic tropical soils.

## Methods

### Batch experiments

Batch experiments were conducted in replicates using a series of 30 ml well-mixed suspensions of CA (Sigma, purity ≥98%) and kaolinite (0.15 g) in a 0.1 NaCl solution with and without 1 mM FeCl_3_ (kaolinite-Fe(III)-CA system vs kaolinite-CA system) at an initial pH 3.5 13,15. The relatively high Fe^3+^ (1 mM) concentration compared to natural tropical soils (several to hundreds of μM) was chosen in order to satisfy the requirements of the Fe L_2,3_-edge Q-XANES measurements for sorption products, i.e. to obtain spectra with acceptable signal-to-noise ratios. Correspondingly, the concentrations of CA were 0.1, 0.25, 0.5, 1, 2, 4, 8, 12, 16 and 20 mM for batch experiments to ensure a Fe/CA molar ratio in the range of 0.05 to 10, which represent a range of Fe/CA molar ratios found in soil solution of forest and arable soils^14^,^54^,^56^. A binary kaolinite-Fe(III) system without CA addition was also included in this study. The suspensions were shaken for 12 h on an end-over-end shaker in a temperature-controlled room at 4 °C, and then centrifuged to separate the solid phase (sorption samples) and supernatants. The amounts of CA and Fe retained in the sorption samples were calculated by subtracting the concentrations in the supernatant from their initial concentrations. More details about the batch experiments are provided in Supplementary Information (SI).

### Fe K-edge EXAFS spectroscopy

Fe K-edge EXAFS measurements were conducted at beamline X11A at the National Synchrotron Light Source and HXAM/SXRMB beamlines at the Canadian Light Source (CLS, Saskatoon, Canada). A monochromator using a pair of Si(111) crystals was detuned by 40% to suppress high order harmonic X-rays. Reference samples including ferrihydrite (two-line), goethite, Fe(III) citrate (powder) and Fe(III) adsorbed on CA coated kaolinite were used for the EXAFS experiments. Sorption samples (paste form) were selected from kaolinite-Fe(III) system (S0) without CA and kaolinite-Fe(III)-CA systems with various Fe/CA molar ratios (2:1, S3; 1:1, S4; 1:2, S5; 1:4, S6; 1:20, S9). All samples were sealed in thin plastic sample holders using Kapton tape for spectra collection in the fluorescence mode. Fe foil was used for energy calibration at 7112 eV at the three beamlines, and multiple scans (2~6) were conducted for each sample based on the spectra quality. All the Fe EXAFS spectra were processed using Athena and Artemis^57^. Detailed information on data analysis was described in the SI.

### Fe L_3,2_-edge XANES spectroscopy

Fe L_3,2_-edge XANES measurements were conducted at the SGM beamline (11ID-1) at the CLS and Soft X-ray beamline 4B7B at the Beijing Synchrotron Radiation Facility (BSRF). To minimize radiation damage, the established 20-second quick-XANES (Q-XANES) scan mode^58^ was used at the Fe L-edge for the Fe(III) citrate (solid), ferrihydrite (two-line) and selected sorption samples (S0, S3, S6 and S9) except FeCl_3_·6H_2_O, which was collected in normal scan mode at the BSRF where no radiation damage occurred due to the low photo flux^59^. All the samples were mixed with Millipore water and deposited onto Au-coated Si wafers, and air dried for Q-XANES experiments in total electron yield (TEY) mode without self-absorption effects. The beam spot was set to ~10 μm at the SGM beamline. Radiation damage of sorption samples was excluded since there were insignificant changes in peak intensity during continuous multiple (3~11) repeated scans at the same spot on each sample. All Fe L_3,2_-edge XANES spectra were normalized by subtracting the pre-edge intensity at 707.25 eV. The absolute energy scale was set by assigning the energy of the second peak in the 2p_3/2_ signal of Fe to 709.8 eV^58^.

## Additional Information

**How to cite this article**: Yang, J. *et al.* Retention Mechanisms of Citric Acid in Ternary Kaolinite-Fe(III)-Citrate Acid Systems Using Fe K-edge EXAFS and L_3,2_-edge XANES Spectroscopy. *Sci. Rep.* **6**, 26127; doi: 10.1038/srep26127 (2016).

## Supplementary Material

Supplementary Information

## Figures and Tables

**Table 1:** Comparison of the retained citric acid (CA) in the kaolinite-CA and kaolinite-Fe(III)-CA systems as well as the retained Fe in sorption products of the kaolinite-Fe(III)-CA system.

Sorption samples	Initial CA concentration	Initial Fe/CA mol ratio	Retained CA concentration (mg g^−1^kaolinite)		Retained CA/Total CA (%)		Increased CA^a^		
	(mM)		Kaolinite-CA system	Kaolinite-Fe(III)-CA system	Kaolinite-CA system	Kaolinite- Fe(III)-CA system	Concentration (mg g^−1^kaolinite)	percentage %	Retained Fe in kaolinite- Fe(III)-CA system (mg g^−1^ kaolinite)
S1	0.1	10	2.470 ± 0.032	3.842 ± 0.000	64.29 ± 0.90	100.0 ± 0	1.373 ± 0.035	55.58 ± 2.17	10.96 ± 0.00
S2	0.25	4	2.693 ± 0.002	7.355 ± 0.130	28.03 ± 0.03	76.57 ± 1.35	4.663 ± 0.127	173.1 ± 4.52	10.72 ± 0.01
S3	0.5	2	3.474 ± 0.217	10.25 ± 0.229	18.09 ± 1.13	53.37 ± 1.19	6.779 ± 0.011	195.8 ± 11.9	10.44 ± 0.01
S4	1	1	6.137 ± 0.005	15.14 ± 0.107	15.97 ± 0.01	39.40 ± 0.28	9.001 ± 0.102	146.7 ± 1.55	10.02 ± 0.00
S5	2	0.5	12.17 ± 0.326	25.04 ± 0.311	15.84 ± 0.42	32.58 ± 0.40	12.87 ± 0.636	105.9 ± 8.06	9.55 ± 0.09
S6	4	0.25	35.37 ± 3.123	51.92 ± 0.471	23.02 ± 2.03	33.79 ± 0.31	16.55 ± 2.652	47.83 ± 11.7	8.63 ± 0.00
S7	8	0.125	63.14 ± 5.911	79.33 ± 1.371	20.54 ± 1.92	25.81 ± 0.45	16.18 ± 4.540	26.53 ± 9.67	7.29 ± 0.04
S8	12	0.083	103.9 ± 2.342	119.1 ± 2.005	22.53 ± 0.51	25.83 ± 0.45	15.21 ± 4.398	14.75 ± 4.57	6.48 ± 0.00
S9	20	0.050	195.2 ± 12.91	212.0 ± 10.61	25.40 ± 1.68	27.59±1.38	16.82 ± 2.303	8.73 ± 1.76	5.55 ± 0.00

^a^The increased amount of CA retained in the ternary kaolinite-Fe(III)-CA systems relative to the binary kaolinite-CA systems.

**Table 2:** Structural parameters and species of Fe in the selected sorption samples from the kaolinte-Fe(III)-citrate system determined by shell-fitting and linear combination fitting of Fe K-edge EXAFS spectra^a^.

	Shell-fitting												
	Fe-O				Fe-Fe				Fe-Fe				
	d (Å)	CN	σ^2^ (Å^2^)	ΔE1	d (Å)	CN	σ^2^ (Å^2^)	ΔE2	d (Å)	CN	σ^2^ (Å^2^)	ΔE1^d^	R
S0	1.98 (0.01)^b^	5.3^c^	0.010^c^	−3.79	3.13 (0.02)	1.8^c^	0.012	3.06	3.38 (0.05)	2.5^c^	0.010^c^	−3.79	0.0070
	Fe-O				Fe-C				Fe-Al/Si				
	d (Å)	CN	σ^2^ (Å^2^)	ΔE1	d (Å)	CN	σ^2^ (Å^2^)	ΔE2	d (Å)	CN	σ^2^ (Å^2^)	ΔE2^d^	R
S9	2.00 (0.01)	5.0^c^	0.010	−0.42	3.16 (0.03)	1.0^c^	0.001	12.3	3.40 (0.04)	1.0^c^	0.008	12.3	0.0013
Linear combination fitting													
	Goodness of fit				Percentages (%) of targeted components								
	R factor		χ^2 e^		Ferrihydrite				Kaoline-Fe-citrate complex^e^				
S3	0.042		0.145		83 (±3)^b^				17 (±3)				
S4	0.038		0.142		72 (±3)				28 (±3)				
S5	0.026		0.113		34 (±3)				66 (±3)				
S6	0.028		0.128		15 (±2)				85 (±2)				

^a^S0, S3 to S6 and S9 represent sorption samples without CA and with a ratio of Fe/Citrate acid as 2, 1, 0.5, 0.25 and 0.05 in ternary kaolinite-Fe(III)-CA systems respectively. ^b^Fitting uncertainty; ^c^fixed during the fitting; ^d^The same energy shift as the first or second shell was used for the third shell fitting to reduce the number of fitting parameters; ^e^sorption sample S9 used as a reference; ^e^reduced chi-square.
