# Supplementary material for: Retention Mechanisms of Citric Acid in Ternary Kaolinite-Fe(III)-Citrate Acid Systems Using Fe K-edge EXAFS and L3,2-edge XANES Spectroscopy
Source: Sci Rep. 2016 May 23;6:26127. doi: 10.1038/srep26127 (PMC4876610; doi:10.1038/srep26127)
Supplement: Supplementary Information [file srep26127-s1.docx]

**Supplementary information**

**Retention Mechanisms of Citric Acid in Ternary Kaolinite-Fe(III)-Citrate Acid Systems Using Fe K-edge EXAFS and L_3,2_-edge XANES Spectroscopy**

Jianjun Yang^1*^, Jian Wang^2^, Weinan Pan^1^, Tom Regier^2^, Yongfeng Hu^2^, Cornelia Rumpel^3^, Nanthi Bolan^4,5^, Donald Sparks^1^

1. Department of Plant and Soil Sciences, Delaware Environmental Institute, University of Delaware, Newark, USA, 19716
2. Canadian Light Source Inc., University of Saskatchewan, Saskatoon, Canada, S7N 0X4
3. CNRS, Institute of Ecology and Environment Paris, IEES, UMR (CNRS-INRA-UPMC-UPEC-IRD), Thiverval-Grignon, France, 78850
4. Global Centre for Environmental Remediation (GCER), University of Newcastle, NSW, 2308, Australia
5. Centre for Environmental Risk Assessment and Remediation (CERAR),University of South Australia, Mawson Lakes, Australia, SA 5095

***Corresponding Authors:**

Jianjun Yang, E-mail: jianjun@udel.edu. Phone: (302)7663823, fax: (302)8310605.

**Sample preparation and characterization.**

The two-line ferrihydrite was synthesized according to the method recommended by Cornell and Schwertmann[^1^](#_ENREF_1) and confirmed by XRD analysis. Fe(III) citrate, Fe(II) oxalate and FeCl_3_•6H_2_O were purchased from Sigma. Kaolinite, purchased from Wards Natural Science Establishment Inc., Rochester, N.Y, was ground to pass through a 75-μm sieve and used after removing Fe hydroxides[^2^](#_ENREF_2). The surface area of kaolinite particles (14.55 m^2^ g^-1^) was determined by N_2_-BET surface area analysis.

**Batch experiments**

Stock solutions of FeCl_3_ (5 mM), citric acid ( CA, 30 mM) and background solution of NaCl (0.1 M) were prepared separately, and adjusted to pH 3.0 using 0.1 M HCl and/or NaOH. With a final total solution volume of 30 mL, different volumes of FeCl_3_ and CA stock solution as well as background NaCl solution were added into polyethylene centrifugation tubes according to each Fe/CA molar ratio that ranged from 0.1 to 10. Preliminary experiments indicated 12h was enough for CA to reach equilibrium in the investigated kaolinite-Fe(III)-CA and kaolinite-CA systems. After shaking 12h, the suspensions of the two investigated systems were centrifuged at 12000 rpm for 15 mins. The supernatants were transferred to separate polyethylene centrifugation tubes and then their pH values were measured. The remaining sorption sample in each tube was washed twice by adding 10 mL of a 0.1 NaCl solution and mixing well before centrifugation, and then storaged in a 4 ℃ cooling room. To prepare for the Fe (III) adsorbed on CA coated kaolinite reference for EXAFS measurements, sorption sample S9 (Fe/CA molar ratio = 1: 20) in the kaolinite-CA system were further added in 30 mL 0.5 mM FeCl_3_ solution with pH at 3.0 and centrifuged at 12000 rpm for 15 mins after shaking for 48h. After removing the supernatant, the remaining sorption sample was washed twice by adding 10 mL of a 0.1 NaCl solution and centrifuged again after well-mixing to obtain the sorption sample. Part of each sorption sample (paste form) was directly used for Fe K-edge EXAFS measurements, while some of each sorption sample was freeze-dried for bulk Fe L_3,2_-edge XANES experiments. The CA content in each supernatant was determined by a reversed-phase liquid chromatographic method[^3^](#_ENREF_3). The Fe concentration in each supernatant was determined by inductively coupled plasma atomic emission spectroscopy (ICP-AES).

**EXAFS data analysis**

Energy was set to 7112 eV (E_0_) for each merged EXAFS spectrum using Athena, and the spectra were normalized to unit step height using a linear pre-edge subtraction and quadratic polynomial as the post-edge line to conduct background subtraction. Then the spectra were transformed to k-space based on E_0_. After that, the data were Fourier transformed (FT) using the hanning window to isolate individual frequencies in the χ (k^3^) spectra with a k range of 3.0 to 11 Å^-1^, without correction for phase shift. As a single Fe phase was expected to be present in samples S0 and S9, the shell-fitting analysis of these two EXAFS spectra were conducted using Artemis; the theoretical backscattering phase and amplitude functions for backscatters were calculated by the FEFF6.0 code using structural modes of solution Fe citrate (Fe-C path)[^4^](#_ENREF_4), ferrihydrite (Fe-O path)[^5^](#_ENREF_5) and zeolite containing Fe(III) (Fe-Al/Si path)[^6^](#_ENREF_6). The amplitude reduction factor (S_0_^2^) was set to 0.65, which was determined by fitting the first shell of the ferrihydrite spectra. All the EXAFS fitting was conducted in R space within 0 ~ 3.5 Å. After confirming the presence of Fe(III) as a kaolinite-Fe(III)-citrate complex in sample S9, principle component analysis coupled with targeted analysis were first conducted for the selected sample S0 and S3 to S6, and then linear combination fitting (LCF) was further applied to quantitatively assess the proportion of each significant Fe specie in these samples using ferrihydrite, goethite, Fe(III)-citrate (solid) and sample S9 as reference spectra.

**Additional Tables and Figures.**

**Table S1 Solution pH and soluble Fe and Al concentrations in the kaolinite-CA and kaolinite-Fe(III)-CA systems after 12h batch experiments.**

| Solution  samples | Initial CA concentration | pH | |  | Soluble Fe concentration  (mg L^-1^) | |  | Soluble Al concentration  (mg L^-1^) | |
| --- | --- | --- | --- | --- | --- | --- | --- | --- | --- |
|  | (mM) | Kaolinite-CA system | Kaolinite-Fe(III)-CA system |  | Kaolinite-CA system^a^ | Kaolinite-Fe(III)-CA system |  | Kaolinite-CA system | Kaolinite-Fe(III)-CA system |
| S1 | 0.1 | 3.60±0.07 | 3.60±0.05 |  | - | 1.050±0.000 |  | 2.750±0.000 | 2.725±0.025 |
| S2 | 0.25 | 3.58±0.03 | 3.52±0.01 |  | - | 2.250±0.050 |  | 2.750±0.000 | 2.500±0.050 |
| S3 | 0.5 | 3.65±0.08 | 3.45±0.01 |  | - | 3.650±0.050 |  | 2.775±0.025 | 2.675±0.075 |
| S4 | 1 | 3.58±0.01 | 3.46±0.00 |  | - | 5.750±0.000 |  | 2.900±0.000 | 2.800±0.000 |
| S5 | 2 | 3.59±0.02 | 3.46±0.02 |  | - | 8.085±0.485 |  | 3.000±0.000 | 2.875±0.075 |
| S6 | 4 | 3.60±0.05 | 3.49±0.03 |  | - | 12.68±0.025 |  | 3.225±0.025 | 3.075±0.025 |
| S7 | 8 | 3.65±0.04 | 3.48±0.02 |  | - | 19.38±0.235 |  | 3.450±0.000 | 3.500±0.000 |
| S8 | 12 | 3.64±0.03 | 3.51±0.01 |  | 0.025±0.025 | 23.45±0.000 |  | 3.650±0.000 | 3.800±0.050 |
| S9 | 20 | 3.60±0.04 | 3.50±0.01 |  | 0.050±0.000 | 28.08±0.035 |  | 4.000±0.100 | 4.375±0.025 |

a, the short dash indicates the Fe in the supernatant of kaolinite-CA systems was undetectable.

**Table S2 Increased amounts of citric acid (CA) retained on kaolinite from the batch experiment and the proposed EXAFS-derived molecular model in the kaolinite-Fe(III)-CA system at an Fe(III)/CA molar ratio of 0.05.**

| Sample | Fe content  (mM g^-1^ kaolinite) | Enhanced CA content  (mM g^-1^ kaolinite) | | Recovery  (%) |
| --- | --- | --- | --- | --- |
|  |  | Batch experiment | Molecular Model |  |
| S9 | 0.079 | 0.088 | 0.079 | 89.8 |

**Table S3**. **Results of the first four components obtained from principle component analysis (PCA) on k^3^-weighted Fe K-edge EXAFS spectra (3 ~ 11 Å) of the five studied sorption samples of S0, S3, S4, S5 and S6^a^.**

| Components | Eigenvalue | Variation | Accumulated variation | IND^b^ |
| --- | --- | --- | --- | --- |
| Component 1 | 57.775 | 0.783 | 0.783 | 0.3024 |
| Component 2 | 8.535 | 0.115 | 0.899 | 0.2926 |
| Component 3 | 3.359 | 0.045 | 0.944 | 0.5454 |
| Component 4 | 1.283 | 0.038 | 0.982 | 1.2839 |

a, S0, S3 to S6 represent sorption samples without CA and the ratio of Fe/citrate acid as 2, 1, 0.5 and 0.25, respectively; b, the minimum IND number for component 2 indicates the presence of two significant components.

**Table S4 Results of target transform analysis of the k^3^-weighted Fe K-edge EXAFS spectra (3 ~ 11 Å) of the five studied sorption samples of S0, S3, S4, S5 and S6^a^.**

| Targeted reference | Spoil value^b^ | R factor |
| --- | --- | --- |
| Ferrihydrite | 2.907 | 0.058 |
| Goethite | 4.206 | 0.331 |
| Fe(III) citrate (solid) | 5.314 | 0.124 |
| Fe(III) adsorbed on CA coated kaolinite | 2.990 | 0.074 |
| Kaolinite-Fe(III)-citrate complex (S9) | 1.483 | 0.008 |

a, S0, S3 to S6, S9 represent sorption samples without CA and with the ratio of Fe/citrate acid as 2, 1, 0.5, 0.25 and 0.05, respectively; b, Spoil value < 3 suggests an acceptable target has been identified; spoil value between 3 and 6 suggests a moderately acceptable target; and spoil value > 6 suggests an unacceptable target (Beauchemin et al., 2002).

**Table S5 Averaged content of ternary complexed Fe as kaolinite-Fe(III)-citrate complex and its contribution to the retention of citrate acid (CA) in selected sorption samples obtained from the kaolinite-Fe(III)-CA systems.**

| Samples  (Fe/CA molar ratios)^a^ | Ternary-Fe^b^  (mg g^-1^ kaolinite) | Ternary-Fe  (mmol g^-1^ kaolinite) | Ternary-Fe^c^  (μmol) | Ternary-CA^d^  (μmol) | Percentage of ternary-CA to total retained CA (%) |
| --- | --- | --- | --- | --- | --- |
| S3(2) | 1.774 | 0.030 | 4.555 | 4.555 | 56.89 |
| S4(1) | 2.805 | 0.048 | 7.200 | 7.200 | 60.91 |
| S5(0.5) | 6.303 | 0.108 | 16.18 | 16.18 | 82.75 |
| S6(0.25) | 7.336 | 0.126 | 18.83 | 18.83 | 46.44 |

a, S3 to S6 and S9 represent sorption samples with a ratio of Fe/citrate acid as 2, 1, 0.5 and 0.25 in ternary kaolinite-Fe(III)-CA systems respectively; b, the content of ternary complexed Fe (ternary-Fe) for each sample was equal to the content of the retained Fe (Table 1) multiplied by the percentage of ternary-Fe determined by linear combination fitting of Fe K-edge EXAFS spectra (Table 2); c, the content of ternary-Fe in the investigated kaolinite-Fe(III)-CA systems with 0.15 g kaolinite addition; d, the content (mol) of ternary complexed CA (ternary-CA) for each sample was equal to that of the ternary-Fe according to the molecular model (Figure 2b) derived from shell-fitting of Fe K-edge EXAFS spectra of the S9 sample.

Figure S1 Fourier transform magnitude of k^3^-weighted Fe K-edge EXAFS spectra of the selected sorption samples (a) and Fe references (b). S0, S3 to S6 and S9 represent sorption samples without CA and with the ratio of Fe/citrate acid as 2, 1, 0.5, 0.25 and 0.05, respectively. Dotted lines represent peaks of interest: Fe-O backscatter pair for all samples; Fe-Fe backscatter pair for goethite, ferrihydrite and S0 (See Figure 1b for details); Fe-C and Fe-Al backscatter pair for S9 (See Figure 1b for details); multiple scattering peaks due to the Fe-C-O structure^7,8^ for Fe citrate and Fe(III) adsorbed on CA coated kaolinite.

Figure S2 Crystal field diagrams for five-coordianted Fe(III) with D_3h_ symmetry and six-coordinated Fe(III) with O_h_ symmetry. Peaks 1-2 arose from the transition of Fe 2p_3/2_ electrons to the unoccupied d-orbital of Fe(III) with D_3h_ and O_h_ symmetry, respectively.

Figure S3 Sorption isotherm of citric acid (CA) in kaolinite-CA and kaolinite-Fe(III)-CA systems.

**References**

1 Cornell, R. M. & Schwertmann, U. *The iron oxides: structure, properties, reactions, occurrences and uses*. The 2nd edn, 533 (John Wiley & Sons, 2003).

2 O'Day, P. A., Parks, G. A. & Brown, G. E. Molecular structure and binding sites of cobalt (II) surface complexes on kaolinite from X-ray absorption spectroscopy. *Clays Clay Miner.* **42**, 337-355 (1994).

3 Cawthray, G. R. An improved reversed-phase liquid chromatographic method for the analysis of low-molecular mass organic acids in plant root exudates. *J. Chromatogr. A* **1011**, 233-240 (2003).

4 Matzapetakis, M. *et al.* Synthesis, spectroscopic and structural characterization of the first mononuclear, water soluble iron-citrate complex, (NH_4_)_5_Fe(C_6_H_4_O_7_) _2_ 2H_2_O. *J. Am. Chem. Soc.* **120**, 13266-13267 (1998).

5 Michel, F. M. *et al.* The structure of ferrihydrite, a nanocrystalline material. *Science* **316**, 1726-1729 (2007).

6 Choi, S. H., Wood, B. R., Ryder, J. A. & Bell, A. T. X-ray absorption fine structure characterization of the local structure of Fe in Fe-ZSM-5. *The J. Phys. Chem. B* **107**, 11843-11851 (2003).

7 Karlsson, T. & Persson, P. Coordination chemistry and hydrolysis of Fe (III) in a peat humic acid studied by X-ray absorption spectroscopy. *Geochim. Cosmochim. Acta.* **74**, 30-40 (2010).

8 Karlsson, T., Persson, P., Skyllberg, U., Mörth, C.-M. & Giesler, R. Characterization of iron (III) in organic soils using extended X-ray absorption fine structure spectroscopy. *Environ. Sci. Technol.* **42**, 5449-5454 (2008).
